# Identification of predictors of the ovarian response to clomiphene citrate in infertile women with polycystic ovary syndrome: A *post-hoc* analysis of a randomized controlled trial

**DOI:** 10.3389/fendo.2026.1822007

**Published:** 2026-06-30

**Authors:** Yang Liu, Yi Gong, Jiaxing Feng, Hang Ge, Mengyi Zhu, Hong Yu, Jingshu Gao, Xiaoke Wu

**Affiliations:** 1 The First Affiliated Hospital of Zhejiang Chinese Medical University(Zhejiang Provincial Hospital of Chinese Medicine), Hangzhou, China; 2 Zhejiang Chinese Medical University, Hangzhou, China; 3 Beilun District People’s Hospital, Beilun Branch of the First Affiliated Hospital of Zhejiang University, Ningbo, China; 4 First Affiliated Hospital, Heilongjiang University of Chinese Medicine, Harbin, China

**Keywords:** BMI, clomiphene citrate, ovulation induction, pcos, predictors

## Abstract

**Background:**

Clomiphene citrate (CC) is the first-line medication for inducing ovulation in women with polycystic ovary syndrome (PCOS). However, approximately 20% of patients with PCOS are resistant to CC. This study aims to identify reliable baseline predictors of CC resistance in infertile women with PCOS.

**Methods:**

A *post-hoc* analysis of a large, multicenter randomized controlled trial (PCOSAct trial) conducted in China. The current analysis comprised the 471 participants who were randomized to the active CC arm and completed the requisite follow-up. To identify potential candidate variables, we employed multivariable logistic and LASSO regression analyses. Within the framework of a multivariable logistic regression model, we also estimated the independent associations between the identified candidate variables and resistance to CC. Additionally, we plotted the Receiver Operating Characteristic (ROC) curve and utilized the DeLong method to compare the statistical differences in the area under the curve (AUC). Finally, we constructed a restricted cubic spline (RCS) logistic regression model to illustrate the dose-response relationship between continuous predictor variables and CC resistance.

**Results:**

CC resistance was identified in 32 (6.8%) participants. Body Mass Index (BMI), Total Testosterone (TT), and Anti-Müllerian Hormone (AMH) were useful predictors of ovarian response to CC. The “T+BMI” dual-factor model demonstrated high discriminative power (AUC = 0.801) and was statistically comparable to the three-factor model including AMH (AUC = 0.818; P = 0.347). TT was the strongest individual predictor (OR = 2.73 per 1-unit), while BMI was the most significant modifiable risk factor (OR = 2.49 per 1-SD).

**Conclusions:**

A simplified “T + BMI” assessment provides comparable prognostic utility without the need for AMH testing. For patients at high risk of CC resistance, we recommend upfront use of aromatase inhibitors or low−dose gonadotropins. This strategy avoids ineffective treatment cycles and enables personalized ovulation induction.

**Trial registration:**

The study was registered on ClinicalTrials.gov under the identification number NCT01573858 on July 6, 2012.

## Introduction

Polycystic ovary syndrome (PCOS) is the most common endocrine disorder among women of reproductive age, with an estimated prevalence of 5% to 10% (1). As the leading cause of anovulatory infertility, PCOS accounts for 90–95% of anovulatory cases presenting in fertility clinics (2, 3). Clinically, 70% to 80% of these patients experience chronic anovulation, a condition precipitated by arrested follicular development that fundamentally compromises reproductive potential (4). Typical pathological features of PCOS include arrested follicle development and anovulation, both detrimental to fertility (5, 6). This cessation of follicular growth functions as the underlying mechanism of chronic oligo-anovulation, necessitating effective therapeutic strategies to restore fertility (7, 8).

Clomiphene citrate (CC) remains a first-line pharmacological agent for ovulation induction in clinical practice (9). However, approximately 20% of patients diagnosed with PCOS demonstrate resistance to CC, presenting a significant clinical challenge (10). Existing research indicates that resistance to CC is linked to multiple factors, including metabolic status, hormone levels, and ovarian function (11–16). If healthcare providers could identify resistant patients early in the treatment process, it would allow for considerable time savings and enable the timely provision of alternative therapeutic options. Numerous studies have evaluated various screening characteristics as potential predictors of treatment outcomes with CC. Consequently, this *post-hoc* study aims to delineate specific predictors of the ovulatory response to CC, thereby establishing a clinical framework for personalized therapeutic management.

## Methods and participants

### Study design and participants

This study is a *post-hoc* analysis based on data from the “Acupuncture and Clomiphene for Chinese Women with Polycystic Ovary Syndrome” (PCOSAct) trial, a large, multicenter randomized controlled trial (RCT). The original trial was conducted between July 2012 and November 2014 across 27 hospitals in mainland China, recruiting 1,000 women diagnosed with PCOS according to the modified Rotterdam criteria (17, 18). All patients fulfilled the diagnostic criteria for PCOS according to the modified Rotterdam criteria: oligomenorrhea or anovulation, together with clinical or biochemical hyperandrogenism (modified Ferriman-Gallwey hirsutism score ≥5 in Chinese), and/or polycystic ovaries (PCO). For the present analysis, we included the 471 participants who were randomized to receive active CC and completed the follow-up protocol. All participants provided informed consent, and the study was approved by the institutional review boards of each participating center. The trial was registered at ClinicalTrials.gov (NCT01573858) and the Chinese Clinical Trial Registry (ChiCTR-TRC-12002081) on July 6, 2012 (19). The research conducted in this study received approval from the Ethics Committee of the First Affiliated Hospital of Heilongjiang University of Chinese Medicine (2010HZYLL-010). Eligible subjects were Chinese with PCOS-related anovulatory infertility and were randomly assigned to one of four groups for four consecutive treatment cycles: (1) CC with active acupuncture, (2) placebo with active acupuncture, (3) CC with control acupuncture, or (4) placebo with control acupuncture, receiving therapy for four consecutive menstrual cycles. No enrolled patient was receiving insulin sensitizers or other common PCOS medications.

### Clomiphene citrate treatment

Participants initiated treatment with an oral dose of 50 mg CC (Fertilan, Codal Syntor Ltd, Cyprus) daily from cycle days 3 to 7. The protocol employed a stepwise dose-escalation strategy: if ovulation was not achieved, the daily dose was increased by 50 mg in the subsequent cycle, up to a maximum of 150 mg/day (or 750 mg/cycle). Conversely, if ovulation was confirmed, the effective dose was maintained. Treatment continued for up to four consecutive cycles. Therefore, the fourth cycle followed the same 150 mg daily regimen as the third cycle. Ovulation was defined biochemically as serum progesterone levels exceeding 3 ng/mL. Based on the outcome, participants were categorized as CC Resistant if no ovulation occurred in any of the four cycles, or Non CC-Resistant if ovulation was confirmed in at least one cycle.

### Clinical and laboratory assessments

At the baseline visit, all participants underwent standardized physical examinations conducted by trained staff. Personal histories of participants were collected, and their psychological status was assessed. Comprehensive biochemical tests and anthropometric measurements were conducted on all participants. Blood samples were stored at −20 °C until analysis. The samples were then transported to the central laboratory at Heilongjiang University of Chinese Medicine, an ISO 15189-certified laboratory, for analysis. A pelvic ultrasound examination using a transvaginal probe was performed on any cycle day or specifically during the early follicular phase (days 2–5) of the induced cycle.

### Statistical analyses

All statistical analyses were conducted using R software, with a significance level set at a two-sided P-value < 0.05. For covariates with missing data, multiple imputation was performed using chained equations, generating five complete datasets. Rubin’s rules were applied to combine estimates and standard errors from the imputed datasets. Baseline characteristics between the CC-Resistant and Non CC-Resistant groups were compared. Non-normally distributed data are presented as median with interquartile ranges. Group comparisons were performed using the Wilcoxon rank sum test. Categorical variables were expressed as frequencies (percentages) and compared using Chi‐square test.

Variables showing significant intergroup differences (P < 0.05) or considered clinically relevant were included as candidate predictors. We then used a two-step variable selection strategy. First, least absolute shrinkage and selection operator (LASSO) logistic regression with 10-fold cross-validation was applied to screen and rank candidate predictors. Both lambda.min, which minimizes the cross-validated binomial deviance, and lambda.1se, which represents a more parsimonious model within one standard error of the minimum, were examined to describe the penalized selection process. Second, multivariable logistic regression was performed using the same candidate set to evaluate independent associations with CC resistance. Final predictors were determined by integrating LASSO-based screening, multivariable logistic regression results, biological plausibility, and clinical interpretability, rather than by relying on a single tuning parameter alone.

A final multivariable logistic regression model was constructed with CC status as the binary dependent variable. To present clinically interpretable results, effect sizes for each predictor were reported as odds ratios (ORs) with 95% confidence intervals (CIs) using three complementary scales:

Original Unit Scale: OR per 1-unit increase in the original measurement.
Standardized Scale: OR per 1-SD increase, facilitating comparison of the relative strength of different predictors.
Clinical Increment Scale: OR for pre-specified, clinically relevant increments.

The discriminative ability of the final model was evaluated by calculating the area under the receiver operating characteristic curve (AUC). For the combined diagnosis of two or more indicators, we constructed a multivariate logistic regression model including all selected indicators as independent variables. The predicted probability from this model was used as a composite risk score, and its discriminative ability was assessed by the AUC with 95% CI calculated using the DeLong method.

To describe the dose-response relationship between continuous predictor variables and CC resistance, we constructed restricted cubic spline (RCS) for BMI, TT, and AMH, while adjusting for the other two indicators within each model. Utilizing the median as a pivotal point of reference, we proceeded to plot the OR dose-response curves, which were accompanied by the 95% confidence bands to illustrate the precision of our estimates. Furthermore, we reported the overall association P-value, alongside the non-linear P-value derived from the Wald χ² test. Finally, we performed sensitivity analysis using modified Poisson regression with robust sandwich variance estimators, adjusting for the same covariates as the primary logistic model, to estimate risk ratios (RRs). To assess cross−center generalizability, we conducted a leave−one−center−out (LOCO) internal−external validation. In each iteration, one center was held out as the validation set; the logistic model was refitted using data from the remaining centers, and predictions were generated for the held−out center. Predictions from all centers were pooled to compute the external AUC, Brier score, and calibration metrics.

## Results

### Baseline characteristics

As summarized in **Table 1**, the analysis included 471 Chinese women with PCOS. Based on therapeutic outcomes, 439 participants (93.2%) were classified as Non-CC-Resistant, while 32 (6.8%) were identified as CC-Resistant. Notably, the group identified as CC-Resistant exhibited a higher BMI, a longer duration of attempting to conceive, and exacerbated oligomenorrhea, indicating a more severe impairment of chronic ovulatory function. They also had higher TT and FT, AMH, and FIN, but lower SHBG suggesting a heightened state of hyperandrogenism (HA) and an increased burden of small ovarian follicles. This suggests that CC-Resistant group already exhibited a more adverse metabolic-reproductive endocrine phenotype.

**Table 1:** Baseline characteristics of participants by CC-resistance status.

Category	Characteristic	Group			P
		Total (n=471)	Non CC-Resistant (n=439)	CC-Resistant (n=32)	
Baseline Characteristics & Personal History	Age	28.00(26.00-30.00)	28.00(26.00-30.00)	28.00 (25.00- 30.00)	0.85
	BMI(kg/m²)	23.63 (20.96-26.67)	23.44(20.86-26.36)	28.27 (23.90-30.12)	<0.001
	Time attempting to conceive(month)	24.00 (12.00-30.00)	23.00 (12.00-30.00)	31.00 (12.00- 39.00)	0.01
	Treatment/AC	235 (49.89%)	221 (50.34%)	14 (43.75%)	0.59
	Smoking	8 (1.70%)	7 (1.59%)	1 (3.12%)	1
	Drinking	423 (89.81%)	396 (90.21%)	27 (84.38%)	0.45
	Exercise				0.68
	Daily	70 (14.86%)	64 (14.58%)	6 (18.75%)	
	Weekly	82 (17.41%)	76 (17.31%)	6 (18.75%)	
	1–3 times a month	110 (23.35%)	101 (23.01%)	9 (28.12%)	
	Never	209 (44.37%)	198 (45.10%)	11 (34.38%)	
Hyperandrogenism & Sex Hormone Profile	Hirsutism score	2.00 (1.00-5.00)	2.00 (1.00-5.00)	3.00 (1.00-4.25)	0.4
	Acne score^a^				0.83
	None	324 (68.79%)	300 (68.34%)	24 (75.00%)	
	Mild	111 (23.57%)	104 (23.69%)	7 (21.88%)	
	Light	21 (4.46%)	20 (4.56%)	1 (3.12%)	
	Moderate	12 (2.55%)	12 (2.73%)	0 (0.00%)	
	Severe	3 (0.64%)	3 (0.68%)	0 (0.00%)	
	Acanthosis nigricans score^b^				<0.001
	None	385 (81.74%)	363 (82.69%)	22 (68.75%)	
	Light	74 (15.71%)	66 (15.03%)	8 (25.00%)	
	Moderate	11 (2.34%)	10 (2.28%)	1 (3.12%)	
	Severe	1 (0.21%)	0 (0.00%)	1 (3.12%)	
	T (nmol/l)	1.62 (1.20-2.08)	1.58 (1.17-2.06)	1.98 (1.73-2.45)	<0.001
	SHBG (nmol/l)	34.80 (22.75-53.60)	35.50 (23.05-55.10)	26.05 (19.38-34.50)	0.01
	FT (pmol/l)	2.25 (1.73-2.79)	2.22 (1.69-2.73)	2.82 (2.33-3.33)	<0.001
	LH (IU/l)	9.15 (5.90-13.96)	9.13 (5.86-14.20)	9.61 (7.40-11.77)	0.68
	FSH (IU/l)	6.05 (4.97-7.06)	6.10 (5.03-7.08)	5.72 (4.72-6.29)	0.13
	E2 (pmol/l)	198.70 (156.60-269.80)	197.20 (156.40-273.40)	208.10 (169.85- 241.98)	0.96
	P4 (nmol/l)	1.74(1.2 -2.33)	1.73 (1.25-2.34)	1.77 (1.21-1.96)	0.54
Metabolic Profile	FBG (mmol/L)	4.97 (4.54-5.48)	4.96 (4.54-5.48)	5.00 (4.61-5.29)	0.88
	FIN (μIU/mL)	72.72 (47.26-112.20)	70.86 (45.46 -109.60)	104.75 (68.01-145.17)	<0.001
	HDL (mmol/l)	1.25 (1.00-1.48)	1.25 (1.00-1.48)	1.29 (1.01-1.37)	0.42
	TG (mmol/l)	1.28 (0.93-1.88)	1.25 (0.92-1.87)	1.58 (1.16-2.11)	0.06
	TC(mmol/l)	4.60 (3.95-5.24)	4.57 (3.94-5.21)	4.78 (3.98-6.14)	0.16
Ovarian Function	Antral Follicle Count (Left)	12.00 (12.00-12.00)	12.00 (12.00-12.00)	12.00 (12.00-12.00)	0.22
	Antral Follicle Count (Right)	12.00 (12.00-12.00)	12.00 (12.00-12.00)	12.00 (12.00-12.00)	0.78
	Menstrual cycles(No./y)	6.00 (5.00-8.00)	6.00 (5.00-8.00)	5.00 (3.88-6.50)	0.02
	Duration between menstruation periods(days)	60.00 (45.00-74.00)	60.00 (45.00-72.50)	70.00 (56.25-92.50)	0.02
Psychological Status	SAS^c^	33.00 (29.00-38.00)	33.00 (29.00-38.00)	35.00 (31.0-41.00)	0.22
	SDS^d^	34.00 (29.00-41.00)	34.00 (28.50 -41.00)	36.50 (29.75-43.00)	0.44
Other Biomarkers	AMH (ng/mL)	11.47 (6.89-15.56)	11.10 (6.83-15.41)	15.07 (9.88-19.07)	0.01
	HCY(μmol/L)	7.41 (4.89-10.43)	7.34 (4.89-10.24)	8.08 (5.10-12.40)	0.3

Results are expressed as median (interquartile range) or number (percentage).

BMI, body mass index (calculated as weight in kilograms divided by height in meters squared); T, Total testosterone; SHBG, Sex hormone binding globulin; FT, Free testosterone; LH, Luteinising hormone; FSH, Follicle stimulating hormone; E2, Estradiol; P4, Progesterone; FBG, Fasting glucose; FIN, Fasting insulin; HDL, High-density lipoprotein; TG, Triglyceride; TC, Cholesterol; AMH, Anti-mullerian hormone; HCY, Homocysteine;SAS, Zung Self-Rating Anxiety Scale; SDS, Zung Self-Rating Depression Scale. meter.

a Acne score is based on the classification criteria in the Chinese Acne treatment guidelines.

b Acanthosis nigricans score assessment criteri: Light, The lesion is localized and has a small area (such as in the unilateral neck or armpit); Moderate, Involving symmetrical areas on both sides (such as the neck, armpit, and groin), or spreading outwards; Severe, Extensively affecting the skin folds (such as the neck, armpit, and groin), and even spreading to non-folds areas (such as the face and palm).

c Scores range from 25 to 100, with higher scores indicating more severe Anxiety.

d Scores range from 25 to 100, with higher scores indicating more severe depression.

### Variable selection process for predictors

**Figure 1:**
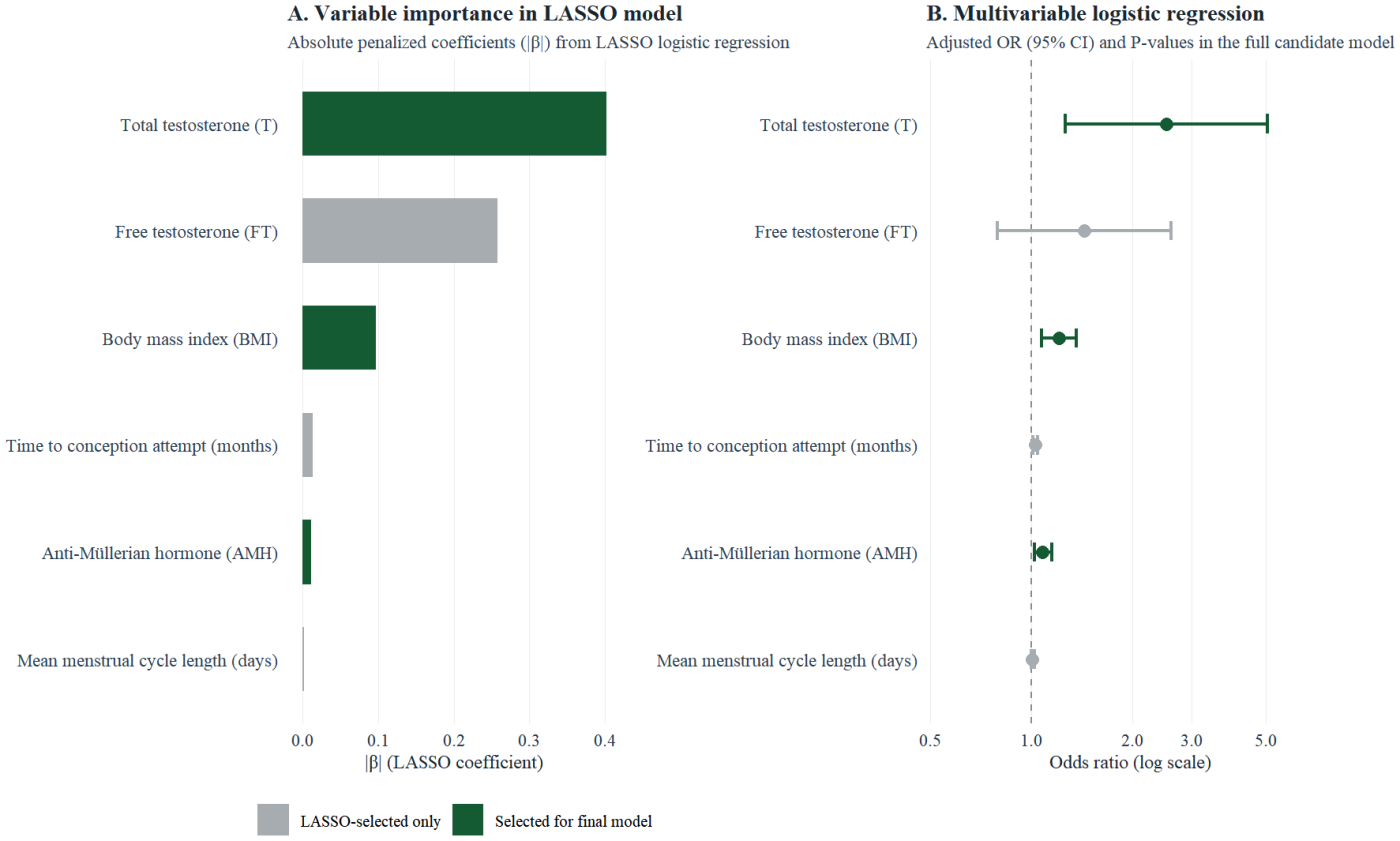
Variable selection process for predictors of CC resistance. **(A)** shows variable importance based on absolute penalized coefficients from the LASSO logistic regression model. **(B)** shows adjusted odds ratios from the multivariable logistic regression model including the candidate predictors. Final predictors were determined by integrating LASSO-based candidate screening, multivariable logistic regression results, biological plausibility, and clinical interpretability. TT, BMI, and AMH were retained as the core predictors for subsequent analyses.

After candidate predictors were identified from baseline comparisons and clinical considerations, we applied LASSO logistic regression and multivariable logistic regression as complementary approaches for variable selection (**Figure 1**). In the LASSO model, candidate variables with non-zero penalized coefficients included testosterone, free testosterone, BMI, time to conception attempt, AMH, and mean menstrual cycle length, suggesting that androgen status, metabolic burden, ovarian reserve, and menstrual irregularity were relevant to CC resistance. In the multivariable logistic regression model including the same candidate set, TT, BMI, AMH, and time to conception attempt showed positive associations with CC resistance. Because TT, BMI, and AMH represented biologically interpretable endocrine, metabolic, and ovarian-response domains, and because they showed consistent statistical support across the LASSO screening and multivariable regression analyses, these three variables were retained as the core predictors for subsequent model construction. Standard LASSO diagnostic plots, including the coefficient path and ten-fold cross-validation curve, are provided in Supplementary.

### Multi-scale effect sizes of the primary predictors

**Figure 2:**
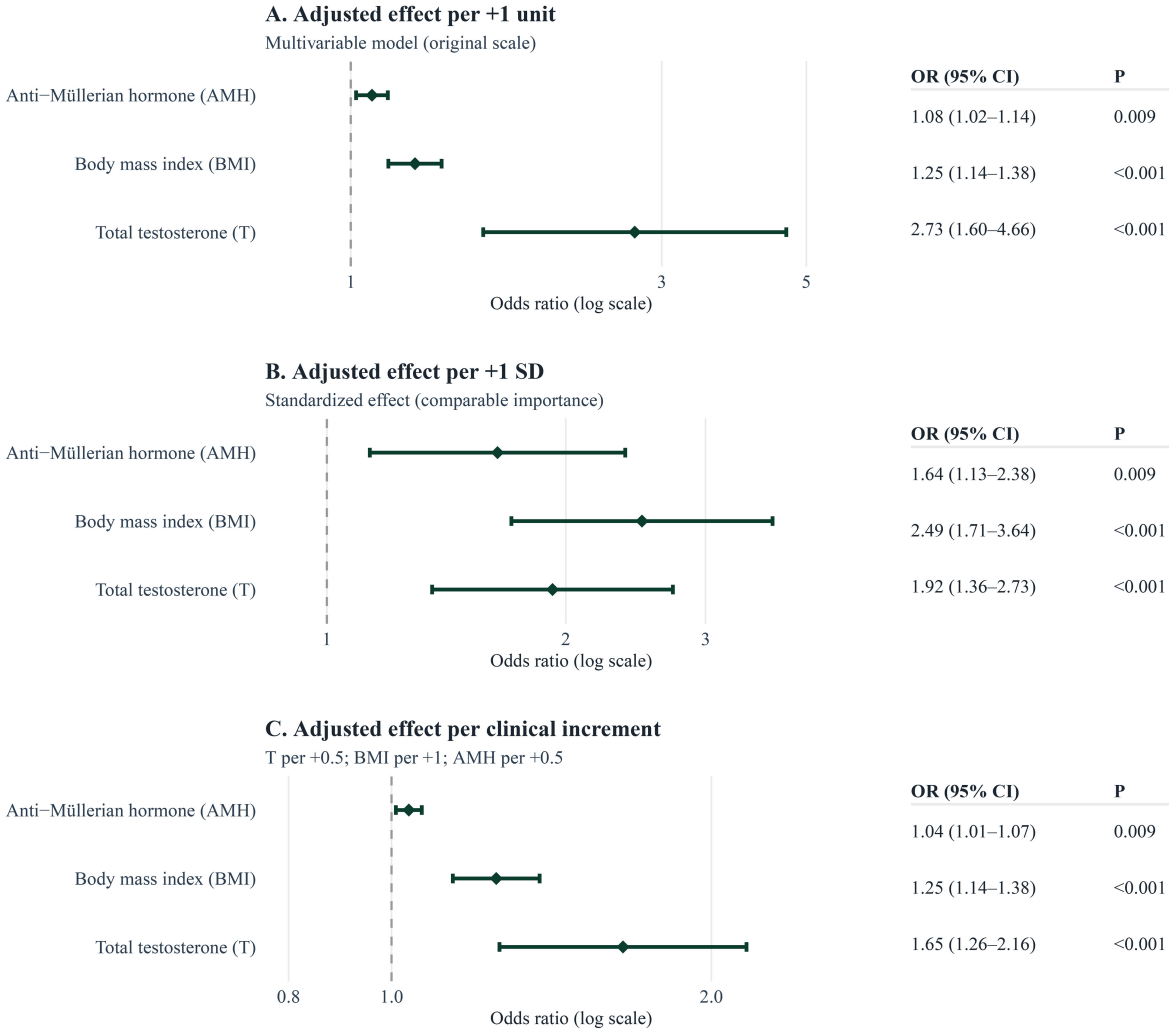
Multi-scale effect sizes of the primary predictors for CC resistance. Forest plots depicting the association (OR) of T, BMI, and AMH with CC resistance, presented on three complementary scales. **(A)** Original Unit Scale: OR per 1 nmol/L (T), 1 kg/m² (BMI), and 1 ng/mL (AMH) increase. **(B)** Standardized Scale: OR per 1-SD increase for each variable. **(C)** Clinical Increment Scale: OR for predefined, clinically relevant increments.

To provide a comprehensive evaluation of these associations, we present effect sizes were analyzed across three scales (**Figure 2**). When considering the original units (Panel A), TT exhibited the strongest effect size. For each 1 nmol/L elevation in TT, the risk of resistance increased nearly threefold (OR 2.73, 95% CI 1.60–4.66). Comparatively, each 1 kg/m² increase in BMI and 1 ng/mL increase in AMH yielded ORs of 1.25 (95% CI 1.14–1.38) and 1.08 (95% CI 1.02–1.14), respectively. When adjusted to reflect an increase of one SD (Panel B), BMI emerged as the paramount predictor of risk variability within the population. A 1-SD increase in BMI (4.03 kg/m²) was associated with the highest OR of 2.49 (95% CI 1.71–3.64), surpassing both TT (0.65 nmol/L; OR 1.92, 95% CI 1.36–2.73) and AMH (6.60 ng/mL; OR 1.64, 95% CI 1.13–2.38). When considering clinical increments (Panel C), which were set to reflect more common clinical variations. An increase of 0.5 nmol/L in TT yielded an OR of 1.65 (95% CI 1.26–2.16), a 1 kg/m² increase in BMI gave an OR of 1.25 (95% CI 1.14–1.38), and a 0.5 ng/mL increase in AMH resulted in an OR of 1.04 (95% CI 1.01–1.07). Notably, this multi-scale analysis provides distinct insights: while TT exhibits the strongest unit-for-unit biological effect, BMI represents the most significant modifiable risk factor for CC resistance. The concurrent elevation of BMI, TT, and AMH thus identifies a high-risk phenotypic profile, highlighting the importance of assessing these parameters for personalized treatment stratification.

### ROC curves

**Figure 3:**
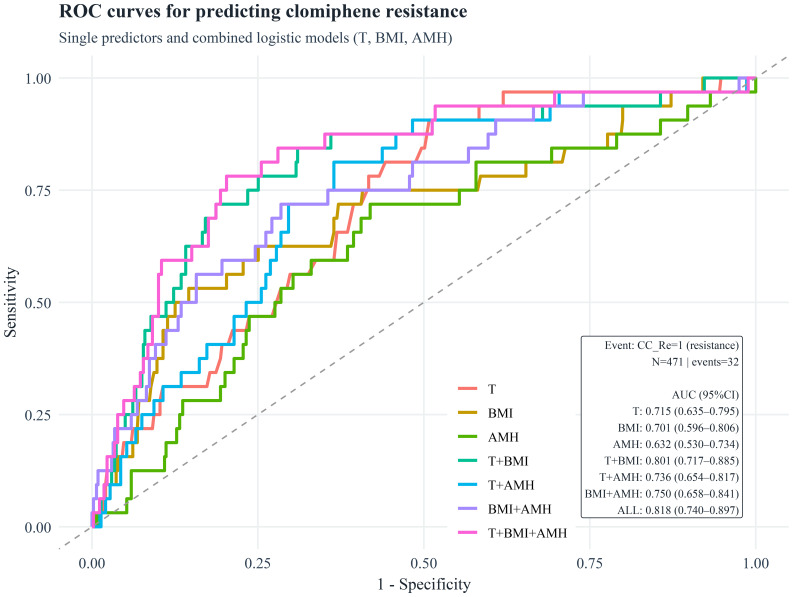
ROC curves comparing the ability of different models to discriminate between CC-resistant and Non-CC-resistant patients.

The discriminative efficacy of individual and combined predictive models was assessed via ROC analysis (**Figure 3**). Among single predictors, TT (AUC = 0.715) and BMI (AUC = 0.701) exhibited significantly superior discriminative ability compared to AMH (AUC = 0.632), establishing them as the primary drivers of the model. In the combinatorial analysis, the dual-factor model integrating TT and BMI (“T + BMI”) achieved an AUC of 0.801, surpassing the threshold for “good” discrimination (AUC > 0.8). This finding underscores that androgenic status and metabolic burden constitute the two fundamental, complementary pathophysiological dimensions governing CC resistance. Statistical comparison using the DeLong test confirmed that the addition of either factor to the other significantly enhanced predictive performance (**Supplementary Table 1**; T vs. T+BMI, Delong P = 0.023; BMI vs. T+BMI, Delong P = 0.011). Although the comprehensive three-factor model (T + BMI + AMH) yielded the highest overall AUC of 0.818, the inclusion of AMH did not provide a statistically significant improvement over the dual-factor model (**Supplementary Table 1**; Delong P = 0.347). Consequently, while AMH demonstrates value when augmenting single-factor models, it offers limited incremental utility once the core metabolic and androgenic parameters are established.

### RCS analysis

**Figure 4:**
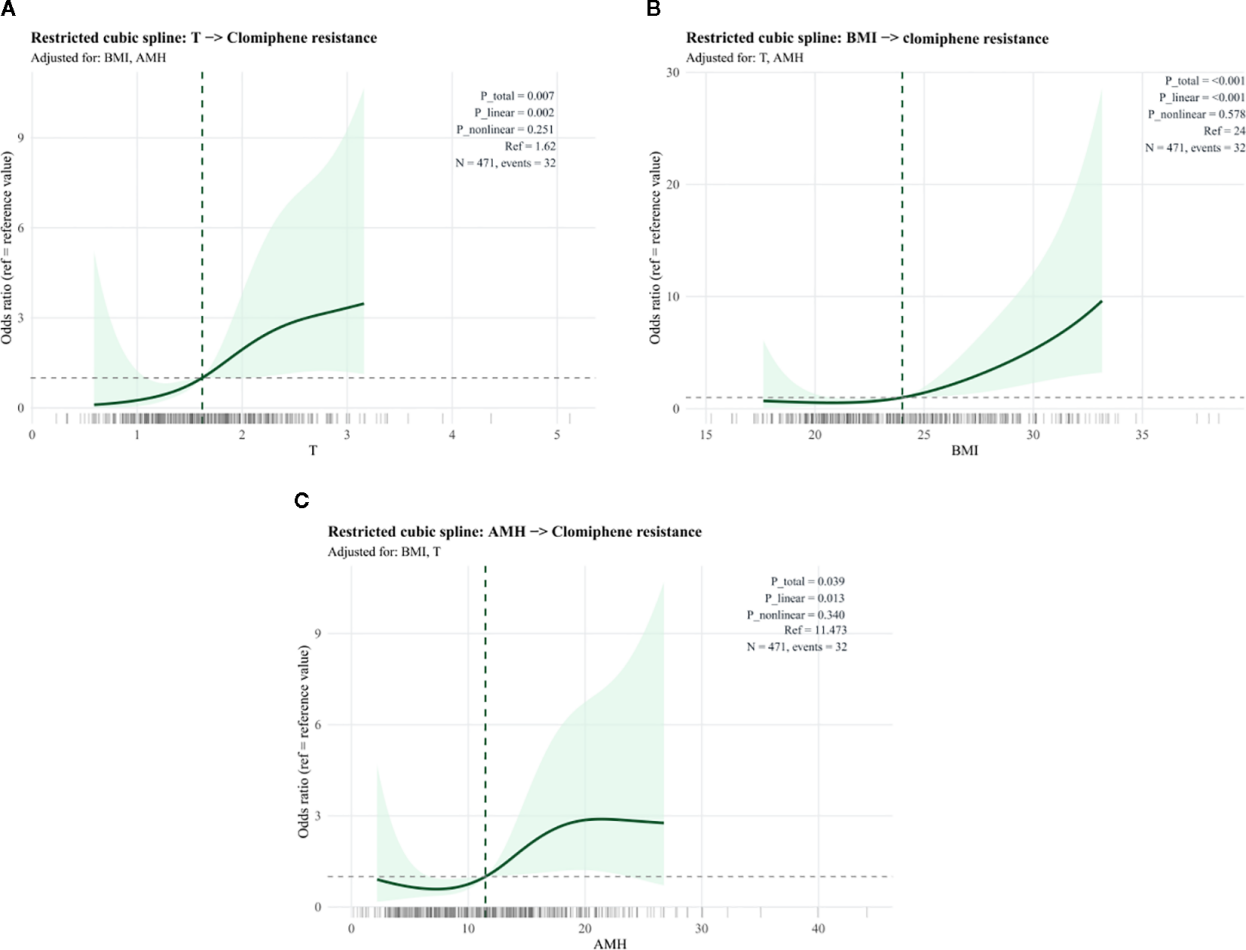
Dose-response relationships between predictors and the risk of CC resistance. **(A)** TT; **(B)** BMI;**(C)** AMH.

RCS analysis was employed to examine the linearity of the association between continuous predictors and the risk of CC resistance (**Figure 4**). Using the median value of 1.62 nmol/L as the reference, TT exhibited a positive linear dose-response relationship (**Figure 4A**; P_overall = 0.007; P_nonlinear = 0.251). The risk of resistance escalated continuously as TT levels increased, with statistically significant deviation from linearity. When considering a BMI of 24 kg/m² as the reference, the risk of resistance rises monotonically as BMI transitions from the normal weight category into the overweight and obese ranges, with a notably steeper slope observed in the higher BMI segments (**Figure 4B**). Furthermore, using an AMH level of 11.47 ng/mL as the reference, it was observed that within the higher range of AMH levels, the risk of resistance significantly increases (**Figure 4C**). In summary, within individuals with PCOS, higher BMI, TT, and AMH levels are clearly correlated with an increased risk of CC resistance, which demonstrates a clear dose-response relationship.

### Sensitivity analysis

In sensitivity analyses using modified Poisson regression with robust standard errors, the estimated associations remained consistent with those from the primary logistic regression (**Supplementary Table 2**). LOCO validation yielded a pooled external AUC of 0.798 and a Brier score of 0.0593, indicating good cross-center generalizability with only minor overfitting (**Supplementary Table 3**).

## Discussion

This *post-hoc* analysis of a large, multicenter RCT underscores the critical role of baseline metabolic and endocrine biomarkers in determining the ovarian response to CC in women with PCOS. We demonstrated that TT, BMI, and AMH serve as independent predictors of CC resistance. Our model comparison revealed that the dual-factor “T + BMI” model (AUC = 0.801) demonstrates predictive efficacy statistically equivalent to the three-factor model (AUC = 0.818). This supports a parsimonious clinical approach: assessing BMI and TT provides a sufficient, accessible, and cost-effective framework for stratifying CC resistance risk, without the need for AMH measurement.

Our results confirm the inverse relationship between serum AMH levels and CC responsiveness, which aligns with AMH’s physiological role as an inhibitor of FSH-dependent follicular recruitment (20, 21). Consistent with the dose-response relationship observed in our analysis, existing literature generally supports AMH as a negative predictor of ovulation. However, the reported predictive thresholds exhibit substantial heterogeneity. Specific cut-off values in previous studies range widely, from as low as 1.2–3.4 ng/mL (16, 22, 23) to as high as 7.77–12.38 ng/mL (11, 12, 24). These variations may be attributed to significant variations in serum AMH levels due to the use of different testing kits, as well as variations in PCOS manifestations and AMH levels among different racial and ethnic groups that likely contribute to these discrepancies. The higher AMH levels observed in our study may be due to our cohort comprising patients from multiple centers nationwide who presented typical cases and were actively seeking infertility treatment. This population represents individuals encountering greater challenges in clinical practice. Individuals with PCOS often have insulin resistance (IR) and high androgen levels. These factors stimulate ovarian cells to produce more androgens, leading to increased small follicle development (23). All these findings support the notion of AMH serves as a surrogate marker for the ovarian small follicle load. It inhibits the aromatase activity of granulosa cells, thereby reducing follicular sensitivity to FSH and diminishing the efficacy of CC in inducing ovulation (25). However, our multivariable analysis reveals that AMH provides limited incremental predictive value. The AUC for AMH alone (0.632) was significantly inferior to that of TT (0.715) and BMI (0.701). Furthermore, adding AMH to the dual-factor ‘T+BMI’ model did not significantly improve discriminative ability. This finding concurs with La Marca et al. (26), suggesting that while AMH reflects ovarian reserve, it is less effective than BMI and TT in stratifying CC resistance risk.

In a prospective observational study of 164 infertile women with PCOS, BMI was identified as a significant predictor of CC resistance, demonstrating an AUC of 0.65 (p=0.001) with an optimal cut-off value of 25.95 kg/m² (15). These findings corroborate our results, which also identified BMI as a primary determinant of CC resistance. Our research team previously discovered that BMI is significantly more effective than other adiposity metrics in predicting ovulation in women with PCOS (27). The current study extends this by quantifying the relative contributions of BMI, TT, and AMH within a unified model, and identifies BMI as the factor with the most substantial effect size (OR = 2.49, 95% CI: 1.71-3.64). Mechanistically, elevated BMI not only increases androgen levels through IR but also directly interferes with the functionality of the hypothalamic-pituitary-ovarian axis, thereby diminishing the pituitary’s responsiveness to CC and reducing FSH secretion (28). This underscores the critical importance of preconception weight management as a first-line intervention that is both key and modifiable to enhance ovulatory function. Legro et al. (29) demonstrated that the prioritization of lifestyle interventions over immediate pharmacological therapy results in significantly higher rates of spontaneous ovulation. Accordingly, both the American Association of Clinical Endocrinologists (AACE) and the American Society for Reproductive Medicine (ASRM) recommend that PCOS patients with a BMI≥25 kg/m² should first pursue weight loss through lifestyle interventions prior to pharmacological treatments (30). Weight loss fundamentally improves the endocrine environment of PCOS by reducing body fat, enhancing insulin sensitivity, and lowering the LH/FSH ratio, thereby enhancing clomiphene’s ovulatory efficacy.

PCOS patients have been shown to experience impaired communication between granulosa cells, which leads to significant dysfunction in both follicle development and ovulation processes (31). A previous study (32, 33) has demonstrated that when the ovarian cortex is incubated with testosterone, it reproduces the changes observed in early folliculogenesis, as reported in various histological studies of PCOS. This finding supports the notion that HA has a detrimental effect on follicular health and ovulatory processes. Imani et al. (34) reported that the Free Androgen Index (FAI) was one of the strongest predictors of ovarian resistance to CC, with 62% of patients exhibiting elevated levels of FAI. Another study (16) suggests that patients with both obesity and hyperandrogenemia are more likely to show resistance to CC therapy. The increased levels of androgens are converted to estrone, which subsequently causes negative feedback on the hypothalamus and pituitary gland, leading to decreased FSH levels and anovulation (35). In our analysis, TT emerged as the most significant individual predictor (OR = 2.73, 95% CI: 1.60-4.66). Furthermore, the high predictive accuracy of the “T + BMI” dual-factor model suggests that BMI and TT represent complementary drivers of CC resistance. This model integrates core pathophysiological markers with clinical accessibility, offering superior prognostic utility compared to single variable models. Elevated androgen levels can downregulate estrogen receptor expression on ovarian granulosa cells, thereby weakening the ability of CC to competitively bind to these estrogen receptors, which ultimately hinders follicle recruitment and dominance. This proposed mechanism offers a potential explanation for the observed association between increased HA and the development of CC resistance in affected patients.

### Strengths and limitations

The main strength of this study stems from its foundation in a large-scale, multicenter RCT, which offers a robust dataset and enhances the generalizability of findings. Nonetheless, several limitations should be acknowledged. First, this research represents a secondary analysis, although we employed multiple imputation to reduce the influence of missing data. Second, We defined CC resistance based on failure to ovulate over four cycles. This is more stringent than the three-cycle threshold used in some clinical guidelines, which may slightly limit the direct extrapolation of results to all clinical settings. Finally, because the present study included all Chinese women with PCOS, the findings might not be generalizable to other populations. In addition, this analysis did not incorporate specific inflammatory markers or genetic polymorphisms, which are possible predictive factors that might have offered more profound understanding into the study’s outcomes.

## Conclusion

In summary, this study demonstrates that a combination of BMI, TT, AMH could be utilized to predict CC treatment response in infertile patients with PCOS. A simplified predictive model incorporating only BMI and TT offers high discriminative accuracy comparable to a model that includes AMH. Notably, although most women had near normal BMI, elevated TT remained an independent predictor of CC non-response. For patients identified as high risk by this model, we recommend avoiding unnecessary CC cycles and instead initiating first line treatment with aromatase inhibitors or, if clinically indicated, low dose gonadotropins. This strategy enables early identification of non-responders, facilitating cost effective and truly personalized ovulation induction.

## Data Availability

The raw data supporting the conclusions of this article will be made available by the authors, without undue reservation.
