# Supplementary material for: Identification of predictors of the ovarian response to clomiphene citrate in infertile women with polycystic ovary syndrome: A post-hoc analysis of a randomized controlled trial
Source: Front Endocrinol (Lausanne). 2026 Jun 30;17:1822007. doi: 10.3389/fendo.2026.1822007 (PMC13364613; doi:10.3389/fendo.2026.1822007)
Supplement: Supplementary file 1 [file DataSheet1.docx]

**Supplemental Material**

**Supplementary table 1. Comparison of AUC among different models**

| Comparison | P_value | P_value_fmt |
| --- | --- | --- |
| T vs (T+BMI) | 0.023348406 | 0.023 |
| BMI vs (T+BMI) | 0.010964531 | 0.011 |
| T vs (T+AMH) | 0.307008248 | 0.307 |
| AMH vs (T+AMH) | 0.012645026 | 0.013 |
| BMI vs (BMI+AMH) | 0.125641029 | 0.126 |
| AMH vs (BMI+AMH) | 0.042730112 | 0.043 |
| (T+BMI) vs (T+BMI+AMH) | 0.347090601 | 0.347 |
| (T+AMH) vs (T+BMI+AMH) | 0.018819393 | 0.019 |
| (BMI+AMH) vs (T+BMI+AMH) | 0.013910214 | 0.014 |

**Supplementary Table 2. Sensitivity analysis of logistic OR and modified Poisson RR estimates across three complementary scales.**

|  |  |  | **Logistic regression** | | **Modified Poisson regression** | |
| --- | --- | --- | --- | --- | --- | --- |
| **Scale** | **Predictor** | **Increment** | **OR (95% CI)** | **P (OR)** | **RR (95% CI)** | **P (RR)** |
| A. Per 1 unit | Total testosterone | per +1 nmol/L | 2.73 (1.60–4.66) | <0.001 | 2.32 (1.59–3.37) | <0.001 |
|  | BMI | per +1 kg/m² | 1.25 (1.14–1.38) | <0.001 | 1.21 (1.13–1.30) | <0.001 |
|  | AMH | per +1 ng/mL | 1.08 (1.02–1.14) | 0.009 | 1.07 (1.02–1.11) | 0.005 |
| B. Per 1 SD | Total testosterone | per +1 SD (0.65) | 1.92 (1.36–2.73) | <0.001 | 1.73 (1.35–2.21) | <0.001 |
|  | BMI | per +1 SD (4.03) | 2.49 (1.71–3.64) | <0.001 | 2.16 (1.64–2.85) | <0.001 |
|  | AMH | per +1 SD (6.60) | 1.64 (1.13–2.38) | 0.009 | 1.53 (1.14–2.05) | 0.005 |
| C. Clinical increment | Total testosterone | per +0.5 nmol/L | 1.65 (1.26–2.16) | <0.001 | 1.52 (1.26–1.84) | <0.001 |
|  | BMI | per +1 kg/m² | 1.25 (1.14–1.38) | <0.001 | 1.21 (1.13–1.30) | <0.001 |
|  | AMH | per +0.5 ng/mL | 1.04 (1.01–1.07) | 0.009 | 1.03 (1.01–1.06) | 0.005 |
| *Note.*  1.Simultaneously adjusted for T, BMI, and AMH (N = 471; 32 events, 6.8%).  2.Modified Poisson regression used robust (sandwich) variance estimators.  3.Across logistic OR and modified Poisson RR models, all three predictors showed fully consistent direction of association and statistical significance (all P < 0.01).  4.Abbreviations: AMH, anti-Müllerian hormone; BMI, body mass index; CI, confidence interval; OR, odds ratio; RR, risk ratio; SD, standard deviation; T, total testosterone. | | | | | | |

**Supplementary Table 3. Sensitivity analysis: Leave-one-center-out internal-external validation of the three-factor model for predicting CC resistance.**

Overall analysis included 471 participants from 21 centers, with 32 resistance events (6.8%).

| **Metric** | **Value** |
| --- | --- |
| Apparent AUC (full sample, training = testing) | 0.818 (0.740–0.897) |
| Pooled LOCO AUC (external validation) | 0.798 (0.717–0.880) |
| Optimism (Apparent -LOCO) | 0.020 |
| Pooled Brier score | 0.0593 |
| Calibration slope (ideal = 1) | 0.863 |
| Calibration intercept (ideal = 0) | -0.305 |
| Centers with evaluable AUC (events ≥ 1) | 17 / 21 |
| Median center-level AUC (IQR) | 0.875 (0.700–0.937) |
| *Note.*  1.LOCO = leave-one-center-out. In each iteration, one center was held out as the validation set, while the remaining centers were used to refit the logistic model.  2.Pooled metrics were computed by aggregating predictions across all held-out centers. This analysis represents center-based internal-external validation rather than validation in an entirely independent external cohort.  3.AUC = area under the receiver operating characteristic curve; CI = confidence interval estimated using the DeLong method.  4.Optimism represents the difference between the apparent AUC and the pooled LOCO AUC.  5.Calibration slope <1 and intercept <0 suggest mild overfitting and a tendency toward risk overprediction.  6.Only centers with at least one resistance event were included in the center-level AUC analysis. | |

| **Supplementary Figure 1. LASSO diagnostic plots for candidate predictor screening.** |
| --- |
| **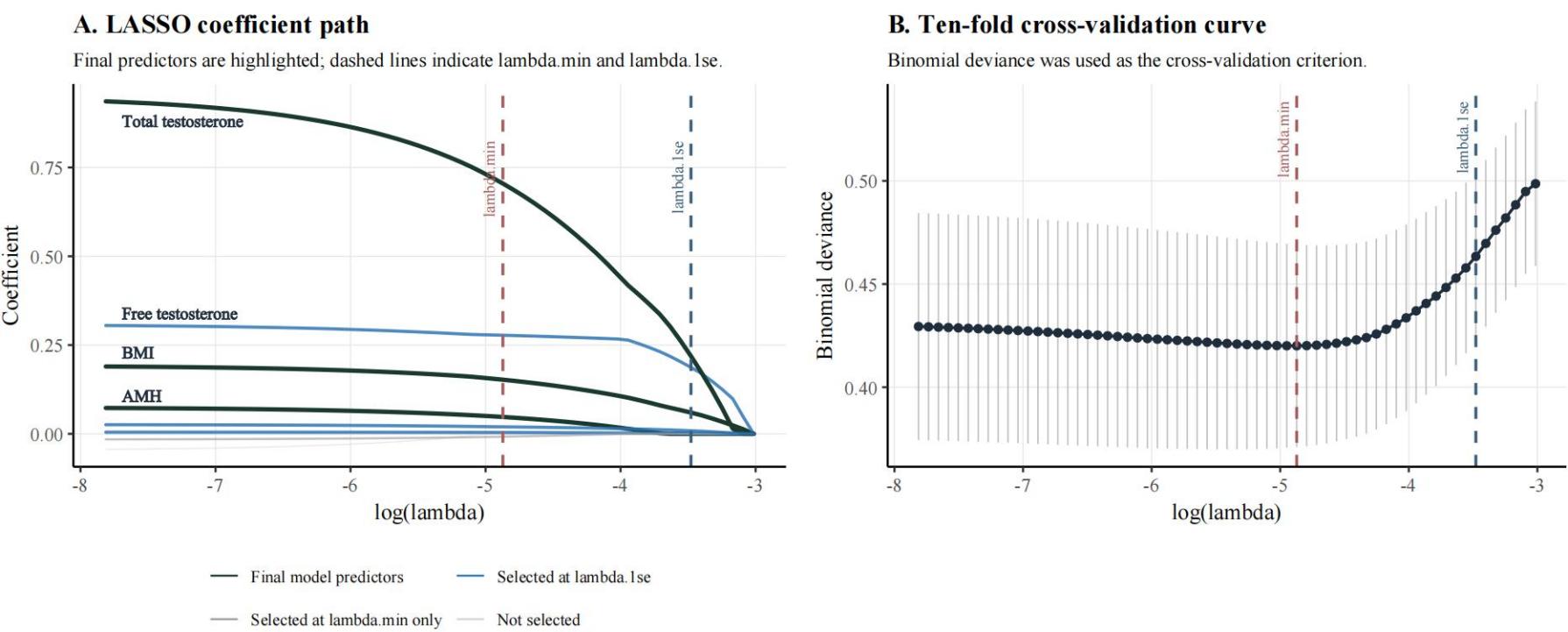** |
| Note.A, coefficient paths of candidate predictors across log(lambda). B, ten-fold cross-validation curve using binomial deviance as the criterion. Vertical dashed lines indicate lambda.min and lambda.1se. These plots were used to visualize the penalized selection process. Final predictors were not determined by LASSO alone, but by integrating LASSO screening, multivariable logistic regression, biological plausibility, and clinical interpretability. |
